# Clinicopathological features and management of colonic lipomas: Case reports

**DOI:** 10.1097/MD.0000000000029004

**Published:** 2022-03-11

**Authors:** Ergin Erginoz, Server Sezgin Uludag, Gokce Hande Cavus, Kagan Zengin, Mehmet Faik Ozcelik

**Affiliations:** a Department of General Surgery, Istanbul University Cerrahpasa – Cerrahpasa School of Medicine, Istanbul, Turkey; b Department of Pathology, Istanbul University Cerrahpasa – Cerrahpasa School of Medicine, Istanbul, Turkey.

**Keywords:** colonic lipoma, lipomatous lesion, mesenchymal tumor

## Abstract

**Introduction:**

Colonic lipomas are benign tumors of adipose tissue that are often asymptomatic, but they may present with rectal bleeding or obstructive symptoms. These tumors are unique in that they are rarely encountered within the gastrointestinal system and can mimic malignant tumors in appearance. Surgical resection and endoscopic removal of tumors have been shown to be successful in their management.

**Patient concerns:**

In this report, we present 3 cases of colonic lipomas, 2 of which are located in the cecum and the other within the sigmoid colon. The presenting symptoms of the patients included abdominal pain, constipation, and dyspepsia.

**Diagnosis:**

Patients typically presented with anemia and an elevated C-reactive protein count. Colonoscopic and computerized tomography findings were used for diagnosis.

**Interventions:**

Hemicolectomy was performed, depending on the localization, and the pathologic specimens were consistent with lipoma.

**Outcomes:**

Surgical resection was curative in all patients. The postoperative period was uneventful in all patients and all patients are symptom-free and alive at 3 years follow-up.

**Conclusion:**

Colonic lipomas are benign mesenchymal tumors of the gastrointestinal system with a male predominance and are observed within the fourth to sixth decades of life. Various genetic abnormalities have been reported and they have been linked to the formation of intussusception. The squeeze sign on radiological imaging, cushion sign and tenting sign in colonoscopy, and naked fat sign during pathologic examination is helpful towards reaching a diagnosis. Surgical resection is the treatment of choice but minimally invasive endoscopic approaches have also been shown to be successful.

## 1 Introduction

Lipomas are benign mesenchymal tumors of the subcutaneous tissue composed of adipose tissue encapsulated by a fibrous layer.^[1]^ Within the gastrointestinal system, they are most likely to be found within the small bowel, stomach, and esophagus.^[2]^ Lipomas are often discovered incidentally and patients become symptomatic when the tumor causes obstruction, leading to abdominal pain, and becomes eroded, leading to bleeding within the bowel.^[3]^

Since lipomas are entirely composed of fat density without any solid component, their radiographic features make them easy to diagnose on imaging modalities such as computerized tomography (CT) and magnetic resonance imaging (MRI). Increased signal intensity on T1- and T2-weighted MRI imaging and low signal intensity on fat-suppressed T2-weighted imaging are supporting evidence towards the diagnosis of a lipoma.^[4]^ A solid component within the mass should raise a concern for the diagnosis of a possible liposarcoma, which is the main disease to be considered in the differential diagnosis.^[5]^ Benign polyps such as an adenoma or malignant lesions such as an adenocarcinoma should also be included in the differential diagnosis.

Treatment of lipomas is often unnecessary and is usually reserved for symptomatic cases. Various surgical resection procedures are available and recently endoscopic removal has also been shown to be effective.^[6]^ In this case series, we present 3 patients with symptomatic colonic lipomas and discuss the relevant literature.

## 2 Methods

The colonic resection specimens were fixated in formalin. The specimens were prepared for histopathological examination with the standard paraffin technique and routine hematoxylin & eosin staining. CDK4 (RTU, Ventana) was used as the primary antibody in the immunohistochemical study. Ethical approval was not obtained since this study involved case reports. Informed consent was obtained from the patients for publication of the case details and accompanying images.

## 3 Case presentations

### 3.1 Case 1

**Figure 1:**
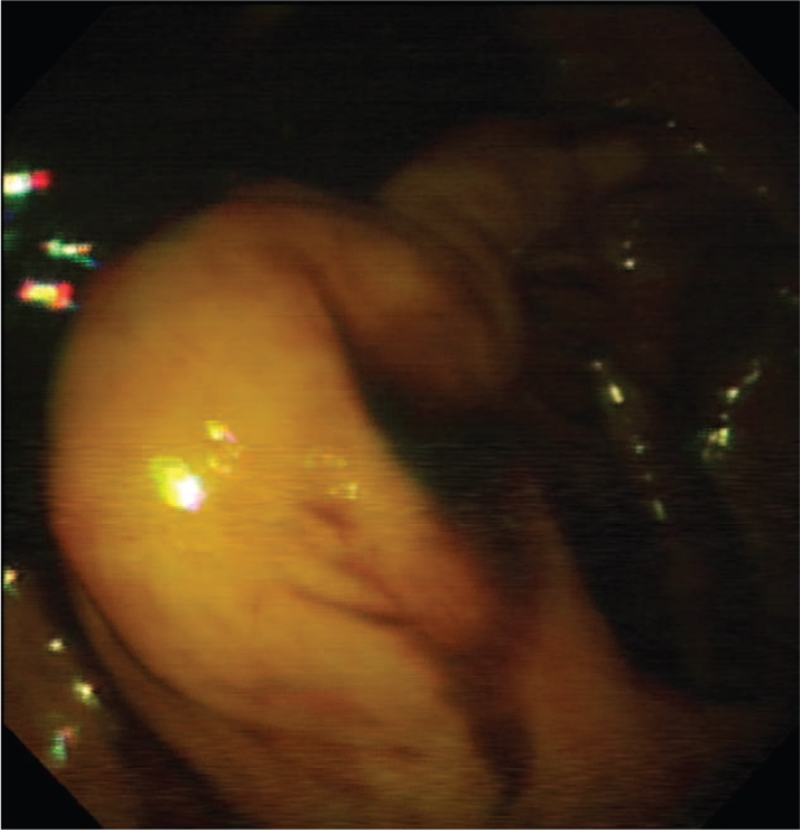
The colonoscopic examination of the lipoma protruding inside the lumen.

**Figure 2:**
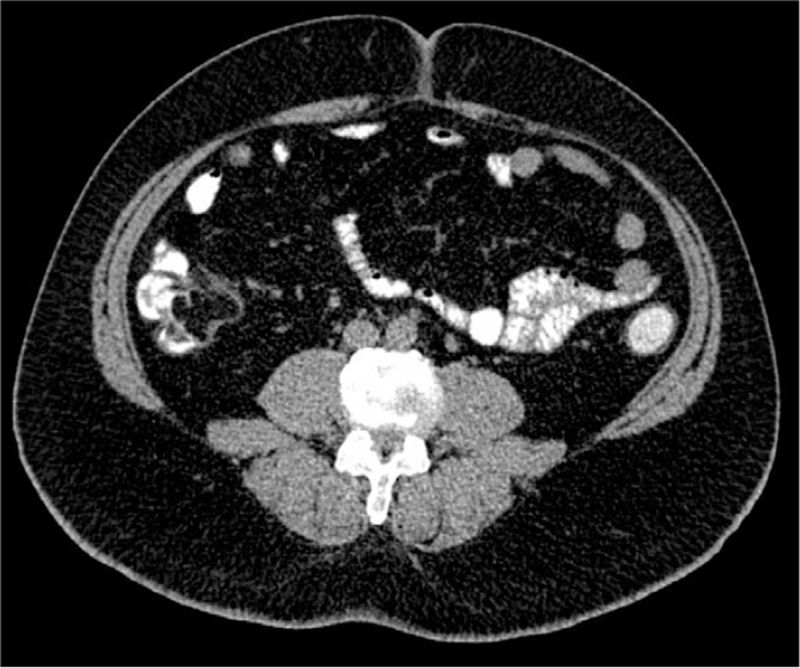
The computerized tomography of the patient showing a mass within the cecum.

A 54-year-old male patient presented to the outpatient clinic with a 1-year-long duration of abdominal pain in the right lower quadrant. Past medical history was remarkable for chronic anal fissure and hepatosteatosis and the past surgical history included laparoscopic cholecystectomy. The laboratory parameters were normal except for a low white blood cell count (4100/μL), low hemoglobin (11.8 g/dL), low platelet count (131,000/μL), and an elevated C-reactive protein (102.2 mg/L). Further work-up of the patient included a CT that showed a non-obstructive mass within the cecum and colonoscopic examination which revealed a lipomatous lesion located on the ileocecal valve (Figs. 1 and 2). A right hemicolectomy was performed and the pathological report was consistent with a lipoma of 6 × 3 × 1.5 cm in size. The postoperative course was uneventful and the patient is symptom-free and alive at 2 years of follow-up.

### 3.2 Case 2

**Figure 3:**
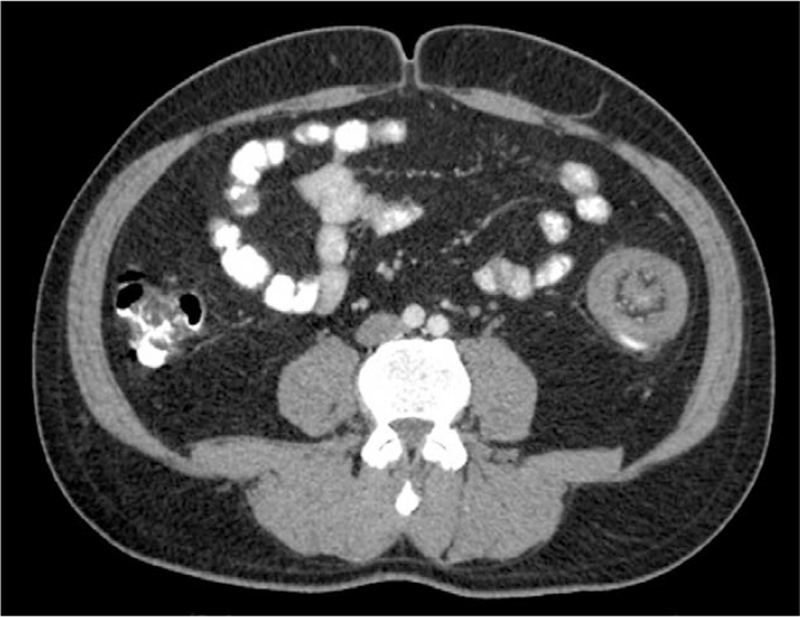
The computerized tomography showing colocolic intussusception within the sigmoid colon.

**Figure 4:**
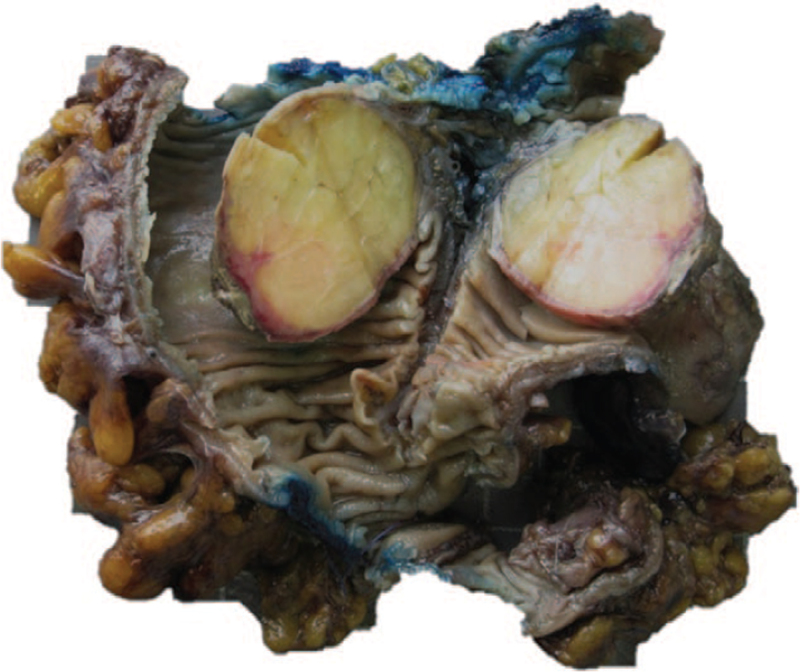
Nodular mass is consistent with adipose tissue when cut open.

A 48-year-old male patient presented to the clinic with a 2-month duration of abdominal pain. The patient denied any accompanying symptoms. Past medical history and past surgical history were unremarkable. Laboratory parameters were normal except for low hemoglobin (12.4 g/dL) and a high C-reactive protein (25 mg/L). The CT showed a lipomatous non-obstructive 4 × 3 cm mass within the sigmoid colon leading to a nearly 10 cm colocolic intussusception (Fig. 3). Laparoscopic left hemicolectomy was performed and the pathological report was consistent with a 4 × 3 cm lipoma (Fig. 4). Postoperative course was uneventful. The patient is symptom-free and alive at 3 years of follow-up.

### 3.3 Case 3

**Figure 5:**
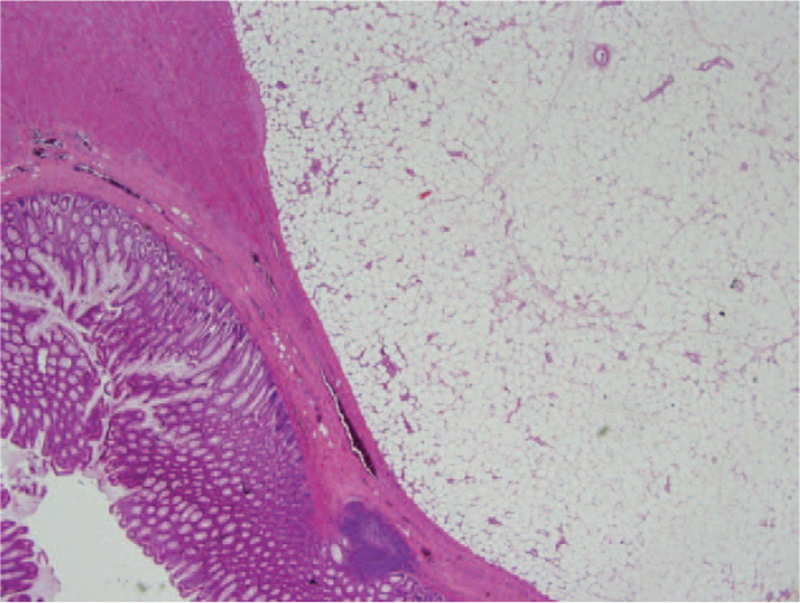
Well-circumscribed lipomas are located in the submucosal and muscular layers. The lipomatous lesions display uniform mature adipocytes without cytological atypia.

A 63-year-old female patient presented to the outpatient clinic with dyspepsia and constipation of 6 months in duration. Past medical history included hemorrhoids and the past surgical history was unremarkable. The laboratory work-up revealed a low hemoglobin count (8.4 g/dL) and an elevated C-reactive protein (71.5 mg/L). A non-obstructive 4 × 2.5 × 1.5 cm mass was located in the cecum and a right hemicolectomy was performed. The pathological report was consistent with a lipoma (Fig. 5). The postoperative course was uneventful and the patient is symptom-free and alive at 2 years of follow-up.

In all of the cases, lesions were located in the submucosal region composed of mature lipocytes. Necrosis and atypia were not observed in any of the cases. In the immunohistochemical study performed with CDK4, no staining was detected in tumor cells.

## 4 Discussion

Lipomas are the most common benign mesenchymal tumors that are observed anywhere in the body and are rarely encountered within the gastrointestinal system. They have a higher tendency to occur in men and usually arise between the fourth and sixth decades of life.^[2]^ The exact pathophysiology of subcutaneous lipoma formation is still not known. However, theories showing a link between soft tissue trauma and lipoma formation, either through the formation of pseudolipomas by prolapsing adipose tissue or through preadipocyte proliferation by cytokine release, have been previously described.^[7,8]^

Several genetic abnormalities have been identified in the formation of lipomas. These include mutations in chromosome 12q13-15, deletions of 13q, and rearrangements of 6p21-33.^[2]^ They are also known to be associated with several genetic disorders such as multiple hereditary lipomatosis, adiposis dolorosa, Gardner syndrome, and Cowden syndrome.^[9,10]^ It is essential to differentiate lipomas from liposarcomas, as the latter poses a threat to life, and this is accomplished by the amplification of the murine double minute-2 gene observed within the malignant liposarcoma.^[11]^

When lipomas become symptomatic, they often bleed or lead to obstruction. In some cases, it may lead to constipation. As observed in the 3 cases presented earlier, abdominal pain is a commonly encountered clinical finding. A common complication of colonic lipomas is that they may lead to colocolic intussusception, as it is widely reported in the literature.^[12–15]^ Intussusception due to a colonic lipoma usually occurs between ages 40 and 70, observed in lesions >4 cm in diameter, and it is more frequently encountered in women.^[12,13]^ Their treatment usually involves surgical resection.

Various diagnostic features aid in diagnosing colonic lipoma. A radiological pathognomonic sign of a colonic lipoma is the squeeze sign, in which a radiolucent filling defect with visible margins is shown to change size and shape due to peristaltic activity when administering barium enema.^[16,17]^ The cushion sign, or the pillow sign, occurs when pressing the forceps on the lipomatous tumor leads to pillowing.^[16]^ The tenting sign occurs when grabbing a pulling a part of the tumor leads to a tent-like appearance. Finally, the naked fat sign is the finding of mature fat cells in the biopsy specimen of the lipomatous tumor.^[2]^

Surgery is the basis of therapy for colonic lipomas, and important advancements in endoscopic approach have emerged. Surgical resection is the treatment of choice for sessile lipomas, lipomas with a diameter >4 cm, lesions with an unclear preoperative diagnosis, lipomas with limited peduncles, giant lipoma leading to intussusception or obstruction, presence of serosa or muscularis propria extending into the pedicle, and failure to perform endoscopic resection.^[18,19]^ Hemicolectomy, segmental colonic resection, or local excision are the options of choice for surgical resection. An intraoperative frozen section is usually needed to ensure negative surgical margins and limit the excision of the tumor to a confined area, which can then eliminate the need for a more radical procedure such as hemicolectomy.^[19]^ Endoscopic submucosal resection is another treatment modality that is usually recommended for pedunculated lipomas or lipomas with a diameter <2 cm.^[20,21]^ The endoscopic submucosal resection method allows for a complete en bloc resection, however, it takes longer to perform and is associated with higher rates of bleeding and perforation.^[22]^ Although Katsinelos et al^[23]^ support the idea that endoscopic removal of lipomas >2 cm in diameter is associated with colonic perforation and hemorrhage due to fatty tissue being an inefficient conductor for electric current, advances in endoscopic techniques led to the use of endoscopic resection even in tumors larger than 2 cm.^[21]^ Other possible surgical techniques include the unroofing technique that was first described by Mimura et al^[24]^ and endoscopic ligation of the base of an elevated lipomatous lesion, so-called the endoloop technique, which was described by Hachisu.^[25]^ An advantage of the endoloop technique is that electrocautery is not needed, which eliminates the risk of perforation or hemorrhage.^[21]^ Endoscopic unroofing is another treatment modality, however, this may leave residual tissue that may require additional resection.^[26,27]^ Only the upper half of the tumor is cut open with the unroofing technique and the lipomatous lesion is propelled outwards towards the opening, which then results in a scarred mucosa.^[21,28]^ Complete removal of the tumor, either surgical or endoscopic, shows favorable outcomes with no known recurrence.

## 5 Conclusion

Colonic lipomas are benign mesenchymal tumors that are often diagnosed incidentally. Most lesions are small and asymptomatic, however, larger lesions may lead to bleeding or cause obstructive symptoms such as abdominal pain. Various diagnostic modalities such as CT or MRI can be used in diagnosis. Resection of the tumor, either surgical or endoscopic, is the treatment of choice and result in a good prognosis if the tumor is removed completely.

## Author contributions

**Conceptualization:** Ergin Erginoz, Server Sezgin Uludag, Mehmet Faik Ozcelik.

**Data curation:** Ergin Erginoz.

**Formal analysis:** Kagan Zengin.

**Investigation:** Kagan Zengin.

**Methodology:** Server Sezgin Uludag, Gokce Hande Cavus.

**Project administration:** Server Sezgin Uludag.

**Resources:** Ergin Erginoz.

**Supervision:** Kagan Zengin, Mehmet Faik Ozcelik.

**Validation:** Gokce Hande Cavus.

**Visualization:** Ergin Erginoz, Server Sezgin Uludag, Gokce Hande Cavus, Mehmet Faik Ozcelik.

**Writing – original draft:** Ergin Erginoz, Gokce Hande Cavus.

**Writing – review & editing:** Ergin Erginoz, Gokce Hande Cavus.
